# Optimization of the Critical Parameters of the Spherical Agglomeration Crystallization Method by the Application of the Quality by Design Approach

**DOI:** 10.3390/ma11040635

**Published:** 2018-04-20

**Authors:** Orsolya Gyulai, Anita Kovács, Tamás Sovány, Ildikó Csóka, Zoltán Aigner

**Affiliations:** Institute of Pharmaceutical Technology and Regulatory Affairs, University of Szeged, 6720 Szeged, Hungary; gyulai.orsolya@pharm.u-szeged.hu (O.G.); anita.kovacs@pharm.u-szeged.hu (A.K.); t.sovany@pharm.u-szeged.hu (T.S.); csoka@pharm.u-szeged.hu (I.C.)

**Keywords:** spherical crystallization, ambroxol hydrochloride, quality by design, factorial design, statistical analysis, powder rheology analysis, spherical morphology

## Abstract

This research work presents the use of the Quality by Design (QbD) concept for optimization of the spherical agglomeration crystallization method in the case of the active agent, ambroxol hydrochloride (AMB HCl). AMB HCl spherical crystals were formulated by the spherical agglomeration method, which was applied as an antisolvent technique. Spherical crystals have good flowing properties, which makes the direct compression tableting method applicable. This means that the amount of additives used can be reduced and smaller tablets can be formed. For the risk assessment, LeanQbD Software was used. According to its results, four independent variables (mixing type and time, dT (temperature difference between solvent and antisolvent), and composition (solvent/antisolvent volume ratio)) and three dependent variables (mean particle size, aspect ratio, and roundness) were selected. Based on these, a 2–3 mixed-level factorial design was constructed, crystallization was accomplished, and the results were evaluated using Statistica for Windows 13 program. Product assay was performed and it was revealed that improvements in the mean particle size (from ~13 to ~200 µm), roundness (from ~2.4 to ~1.5), aspect ratio (from ~1.7 to ~1.4), and flow properties were observed while polymorphic transitions were avoided.

## 1. Introduction

The last step in the production of solid-form active agents and additives is generally crystallization. With the help of crystallization methods, not only can purification be achieved but the ideal crystal habit and polymorphic form can also be produced. With the application of spherical crystallization methods, the flowability and the compressibility properties of the solid materials can be improved [1]. 

The appropriate particle size and powder rheological properties make the material suitable for direct tablet compression [2,3,4]. Direct compression improves the economic aspects by reducing the technological process steps. It is also suitable for processing active substances into solid dosage forms, which is not applicable in the case of wet granulation. Various excipients are used to provide the appropriate dosage form parameters of the tablet in the powder mixture. If the active agent possesses an ideal habit, the amount of the used additives can be reduced. In this way, the production of smaller tablets can be accomplished, which can help patients with dysphagia.

The active pharmaceutical ingredient (API) ambroxol hydrochloride (AMB HCl), which was chosen as the model compound, usually crystallizes in small particles (<20 µm), which causes cohesion between the particles. AMB HCl is the salt form of ambroxol, the metabolite of bromhexine. It is an expectorant with mucokinetic and secretolytic properties used in the treatment of the common cold and cough, bronchial asthma, and chronic bronchitis [5]. However, very little research has been published on the crystallization of AMB HCl. Based on the available literature, direct compression is used for making tablets of this compound [6]. The appropriate physicochemical properties of the directly usable material are important. 

Spherical crystallization methods are categorized into typical [7,8] and non-typical methods [9,10]. In our previous work [11], these methods were compared and the non-typical methods such as spherical agglomeration and cooling crystallization were found to be suitable methods for the production of ambroxol hydrochloride spherical crystals. Spherical agglomeration (SA) is usually described as a typical [12,13] method but in our work, it is rather an antisolvent technique, which is non-typical, due to the fact that only one solvent phase is generated. The cooling crystallization method was implemented with the use of an alternating temperature profile [14,15]. It contains cycles of slow heating and cooling in order to standardize the particle size. It can also be combined with antisolvent addition.

Roundness and aspect ratio are two parameters that are able to describe the morphology of the particles. These values equal 1.00 in the case of a sphere, which has ideal flowability. Micrometric properties are very important for describing the compaction properties of the API [16,17]. For direct compression, the optimal value for mean particle size is different for each active agent and additive, and it also has a role in the surface properties of the particles. According to the literature, the mean particle size of a powder with good flowability is around 80–1000 µm [18,19].

In industrial production, it is important to carry out a risk assessment before applying new technologies. Wrongly selected critical parameters can affect the product in a bad way, which can negatively impact the product, resulting in financial loss. The application of the “Quality by Design” (QbD) methodology according to The International Conference on Harmonisation of Technical Requirements for Registration of Pharmaceuticals for Human Use (ICH) Q8 and Q9 guidelines [20,21] is a fairly new approach in the development phase of new pharmaceutical products [22,23,24]. The QbD concept is a systematic process for the assessment, control, communication, and review of risks to the quality of the APIs through the product lifecycle. The QbD concept provides scientific-based product development, which involves the identification of the quality target product profile (QTPP) defining the critical quality attributes (CQAs), critical material attributes (CMAs), and critical process parameters (CPPs), using risk assessment and optimization of data analysis with the use of design of experiments (DoE) [25,26,27]. Based on the ICH Q8 (R2) guideline, the QTPP means the quality characteristics of a drug product that optimally will be achieved to ensure the desired quality—as promised on the label—taking into account safety and efficacy. A CQA is a physical, chemical, biological, or microbiological property that should meet the predefined requirements to ensure the desired product quality. CQAs are usually associated with the active ingredient, excipients, intermediates, and drug product. CQAs of solid dosage forms affect product purity, strength, drug release, and stability. For drug substances, raw materials, and intermediates, the CQAs can additionally include those properties (e.g., particle size distribution, bulk density) that affect drug product CQAs. A CMA is a physical, chemical, biological, or microbiological property or characteristic of an input material that should be within an appropriate limit, range, or distribution to ensure the desired quality of output material. The variability of a process parameter always has an impact on the CQAs. We call the process parameters “CPPs” if they have a direct impact on CQAs; therefore, these should be monitored and controlled in order to produce the desired quality (temperature, cooling rate, rotation speed, feeding rate, etc.). 

Several quality management tools can be found in the ICH guideline Q9, for example, Ishikawa diagram, Pareto analysis, and risk estimate matrix.

Based on the results of the risk assessment, which show the critical parameters of the procedure, a factorial design can be applied. This is a simple method for screening out the optimal parameters [28,29]. Risk assessment aims at identifying which material attributes and process parameters potentially influence the product CQAs. Furthermore, they can help to obtain all of the significant factors that will be subjected to the DoE study to establish product and process design space (DS) [30,31,32,33].

The aim of our present work was to obtain spherical crystals of the chosen API, AMB HCl, by adapting the QbD approach in the development of the spherical agglomeration method. It is another aspect of the application of risk assessment because it is used in the context of process development and not in the design of the final pharmaceutical product. The result is an active pharmaceutical agent with better morphology and improved flow properties, which makes it possible to apply an easier tableting method during the production.

## 2. Materials and Methods 

### 2.1. Materials

AMB HCl was chosen as the model material and was generously supplied by Teva Pharmaceutical Works Ltd. (Budapest, Hungary). As the mean particle size, aspect ratio, and roundness were key parameters for our work, all of these values for the initial material and the target product are shown in Table 1. The values of the parameters of the target product were described based on the literature and self-made pre-experiments.

All of the applied solvents were of analytical grade. Dimethyl sulfoxide (DMSO) was purchased from Scharlau Concept Co., Ltd. (Budapest, Hungary). Ethyl acetate (EtAc) was purchased from VWR International (Budapest, Hungary).

### 2.2. Methods

#### 2.2.1. Risk Assessment

##### Definition of the QTPP and Determination of CQAs, CMAs, and CPPs 

The first step of a QbD approach is defining QTPPs. These are the quality, safety, and efficiency features of a product, for example, the dosage form, the route of administration, etc. The second step is defining the quality attributes of a product. The CQAs are derived from QTPP and prior product knowledge. In the case of pharmaceutical development, the CQA is a physical, chemical, biological, or microbiological property. The next step is to determine the material attributes and process parameters that may influence the CQAs. These factors were determined based on the literature data, pilot experiments, and personal experiences. Pilot experiments are small-volume experiments that are usually based on visual observations of the key parameters, e.g., particle size and flow properties. 

##### Quality Tools

During the risk assessment, two quality tools were applied: Ishikawa diagram and Pareto analysis. Once the predominant causes have been identified, tools such as the Ishikawa diagram can be applied to identify the root cause(s) of a problem. 

Pareto analysis represents the correlations between CPPs and CQAs and it can determine the most critical parameters to which attention must be given during development [34]. This technique helps the identification of the critical parameters that can influence a process [35]. LeanQbD Software (Version 1.3.6., 2014 QbD Works LLC, Fremont, CA, USA) was used for the evaluation of risk severity scores.

#### 2.2.2. Factorial Design

Based on a well-established risk assessment, the dependent and independent variables of the process can be defined. The levels of the factors have to be determined and as a next step, crystallization needs to be carried out with the previously described factors. As a result, dependent variables can be measured. The Statistica for Windows 13 (Palo Alto, CA, USA) program was used for the statistical evaluation. In a factorial experiment (factorial design, FD), all levels (n) of a given factor (m) are combined with all levels of every other factor included in the experiment, and the total number of experiments is “n·m” [36]. In this case, a 2–3 mixed-level factorial design was used, 36 experiments were planned, and, based on the coefficients, polynomial correlations were defined and the significance of the factors was determined.

#### 2.2.3. Crystallization

**Figure 1:**
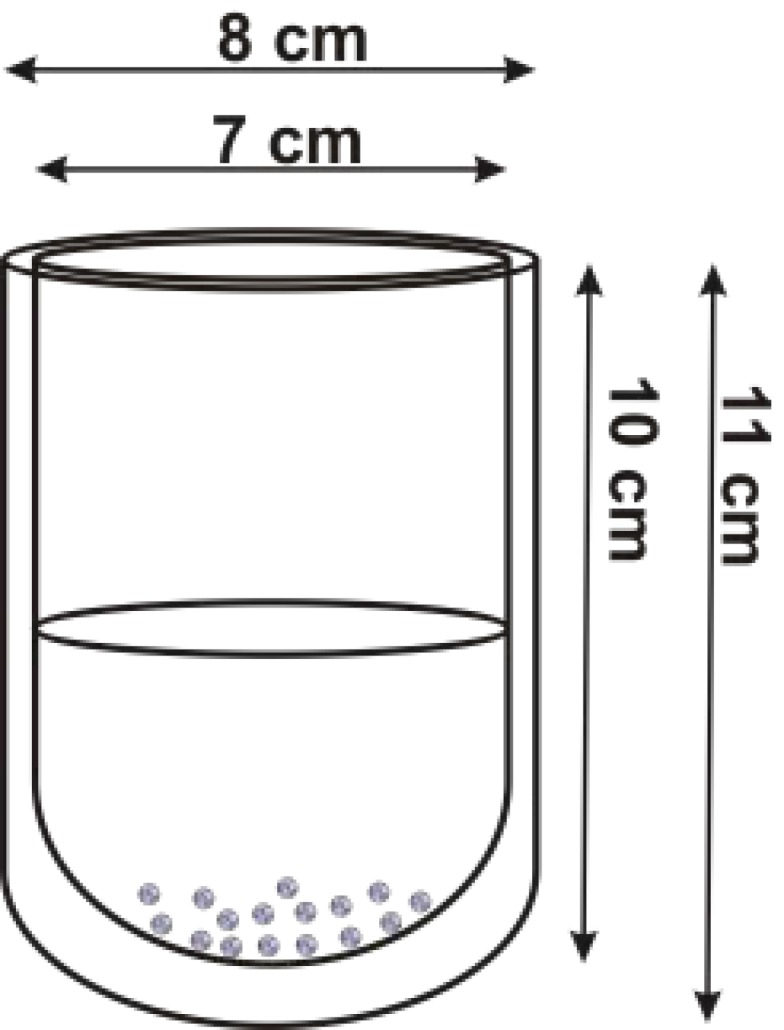
The geometrical parameters of the double-walled vessel used for the production of the spherical agglomerates.

The applied crystallization method was spherical agglomeration, which is an antisolvent technique for the production of spherical crystals. In our case, the antisolvent was ethyl acetate and the solvent for the active pharmaceutical ingredient, AMB-HCl, was DMSO. The saturation of the solution was 440 mg/mL. The temperature difference between the solvents (dT) was set and then the nearly-saturated solution was fed into the antisolvent while agitating the system. Agitation was carried out with a Heidolph Titramax 101 horizontal shaker (Schwabach, St, Louisville, KY, USA) set to 150 rpm. The crystals were produced in a double-walled vessel in the volume of 78 mL and with the exact geometrical parameters shown in Figure 1.

During the crystallization procedure, the temperature was controlled by a Julabo F32-ED Refrigerated/Heating Circulator^®^ (Seelbach, St, Louisville, KY, USA). After crystallization with the chosen parameters, the products were separated by vacuum filtration, washed with 10 mL ethyl acetate, and then dried with a Memmert dry-heat sterilizer (Schwabach, St, Louisville, KY, USA) at 40 °C for 24 h. 

#### 2.2.4. Investigation of the Morphology

The products were examined from the aspect of mean particle size, aspect ratio, and roundness with a LEICA Q500 MC Image Processing and Analysis System (LEICA Cambridge Ltd., Cambridge, UK) measuring an average of 1000 particles. Light microscopic images were also taken, which are shown in the Results section.

#### 2.2.5. Polymorphism

##### X-Ray Powder Diffractometry (XRPD)

Polymorphism was investigated by X-ray powder diffractometry (XRPD) and differential scanning calorimetry (DSC). The diffractograms were collected with a BRUKER D8 Advance diffractometer (Bruker AXS GmbH, Karlsruhe, Germany) system equipped with a Våntec 1 line detector (Bruker AXS GmbH, Karlsruhe, Germany) with Cu Kα1 radiation (wavelength = 1.5406 Å) over the interval 3–40° 2-theta, using 40 kV and 40 mA with rotation switched on. The measurement conditions were as follows: filter, Ni; time constant, 0.1 s; angular step 0.01°, sample holder: Si low background sample holder. 

##### Differential Scanning Calorimetry (DSC)

The DSC curves were collected with a Mettler Toledo DSC 821e apparatus (Mettler Toledo AG, Greifensee, Switzerland) in the temperature interval of 25–300 °C with an Ar gas intake of 10 L/h. Heating was linear with a heating rate of 10 °C/min. The mass of the samples was between 3 and 5 mg in a 40 µL Al sample holder.

##### Fourier-Transform Infrared Spectroscopy (FTIR)

The FTIR spectra of the KBr pastilles of the original AMB HCl and our spherical particles were recorded with an Avatar 330 Thermo Nicolet FTIR spectrometer (Thermo Fisher Scientific Inc., Waltham, MA, USA) between 4000 and 400 cm^−1^ with 128 scans at an optical resolution of 4 cm^−1^.

#### 2.2.6. Powder Rheology

Flow time, angle of repose, and bulk density were measured with a Pharma Test PTG-1 powder characterization instrument (PHARMA TEST Apparatebau AG, Hainburg, Germany) with a Teflon funnel. Tapped density was determined by STAV 2003 Stampfvolumeter (J. Engelsmann AG, Ludwigshafen, Germany) and based on the results, the Carr index and the Hausner factor were calculated. 

#### 2.2.7. Mechanical Strength Tests

The mechanical strength of the crystal agglomerates was numerically characterized by an individually-developed mechanical strength tester apparatus [37]. This instrument measures the force applied for the fracture of the particles or pellets that are put under the breaking head. Each product was tested by breaking 20 individual particles and by calculating the mean fracture force. The software can also represent fracture curves.

## 3. Results

### 3.1. Risk Assessment

#### 3.1.1. Ishikawa Diagram

**Figure 2:**
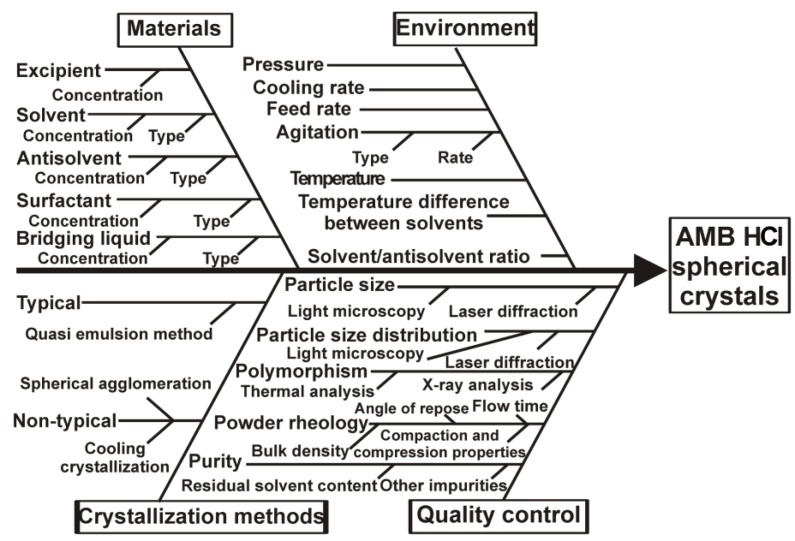
Isikawa diagram for the identification of the key parameters for the production of spherical ambroxol hydrochloride (AMB HCl) agglomerates.

An Ishikawa (fishbone) diagram was constructed to identify the effects of the key material attributes and process parameters on the development of the production of AMB HCl spherical agglomerates with the SA method (Figure 2).

#### 3.1.2. Definition of the QTPPs and Identification of the CQAs

**Figure 3:**
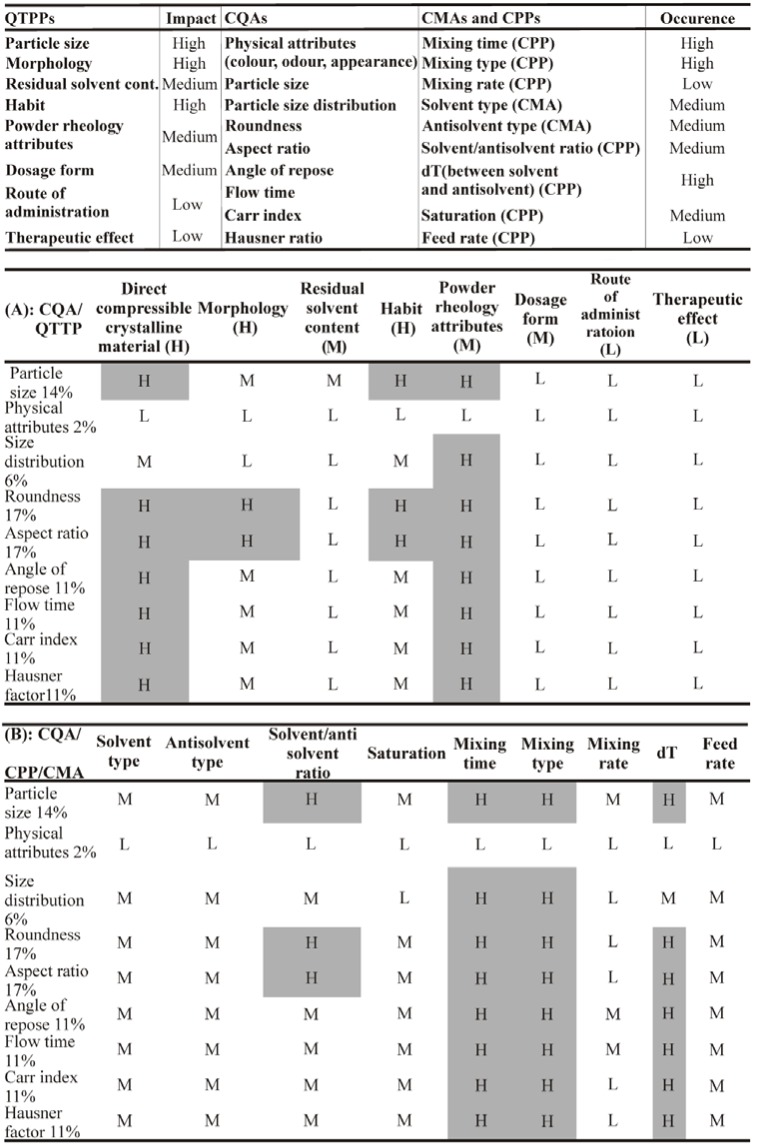
Selected quality target product profiles (QTPPs), critical material attributes (CMAs), critical process parameters (CPPs), and critical quality attributes (CQAs) and their impact on the crystallization system.

Based on the literature data and pilot experiments, QTTPs and CQAs were determined and are shown in Table 2 and Table 3, together with their justification. After the identification of the QTPPs and the CQAs, the following step was to determine the critical material attributes and process parameters (CMAs and CPPs) by risk estimation matrix (REM), which represents the potential risks associated with each material attribute and process parameter that has a potential effect on CQAs. By assigning low (L), medium (M), and high (H) values for each parameter, the REM of interdependence rating between the CQAs and QTTPs was established. For the probability rating, a 1(L)-3(M)-9(H) scale was used. The interdependences of the factors are shown in Figure 3.

Selected QTPPs, CMAs, CPPs, and CQAs and their interdependence rating with the risk estimation matrix (REM): L = low-risk parameter; M = medium-risk parameter; H = high-risk parameter. (A: interdependence between CQAs and QTPPs; B: interdependence between CQAs, CMAs, and CPPs)

#### 3.1.3. Pareto Analysis

**Figure 4:**
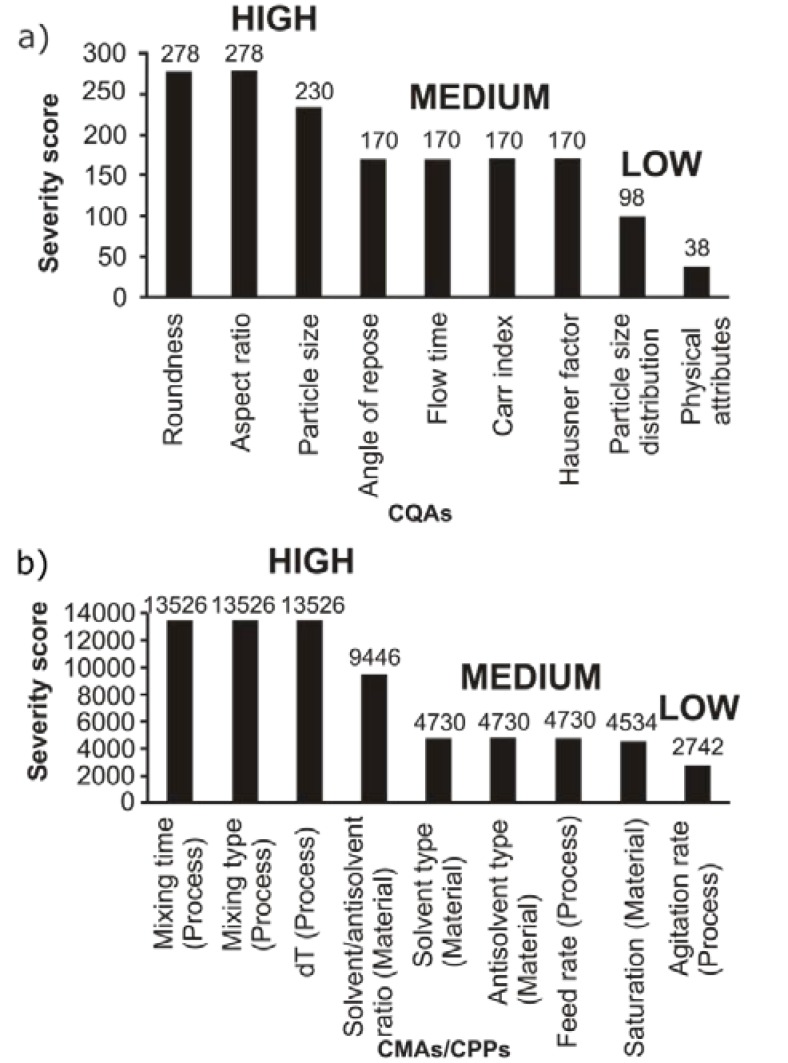
Pareto diagrams of the CQAs (**a**) and the CMAs/CPPs (**b**). Low = low-risk parameter; Medium = medium-risk parameter; High = high-risk parameter.

Based on the REM results, a Pareto chart (Figure 4.) was generated showing the severity scores of CMAs and CPPs. Mixing time and type, the temperature difference between solvent and antisolvent, and the solvent/antisolvent ratio were the factors with the highest severity scores (above 9000). During the following experiments, these factors were the independent variables of the factorial design. The Pareto analysis of CQAs led us to the following conclusions: roundness, aspect ratio, and particle size were the factors with the highest severity scores (above 200) and, according to this, these were the dependent variables of the factorial design in the next step. 

### 3.2. Factorial Design and Crystallization

A mixed 2-3-level factorial design was planned considering the critical parameters of the spherical crystals, which were determined by small-volume pre-experiments and evaluated by LeanQbD™ Software. Mixing type (qualitative factor), mixing time, dT (temperature difference between solvent and antisolvent), and composition (solvent/antisolvent volume ratio) functioned as independent variables. Dependent variables of the factorial design were roundness, aspect ratio, and mean particle size based on the severity scores of CQAs. The levels of the factors are shown in Table 4.

For the factorial design, it was necessary to make 36 products for the examination of the variables, which were as follows: mean particle size, aspect ratio, roundness, and yield. 

Each crystallization was carried out three times in order to investigate the reproducibility and then the relative standard deviation values were calculated, which showed that the methods were reproducible. With the application of Statistica 13 for Windows software, significant factors were determined and are summarized in Table 5. The polynomial functions of the correlations are described, too. In that case, several linear and quadratic factors were neglected in order to improve the adjusted R2; however, decreasing the number of the factors led to a decrement of R2.

**Figure 5:**
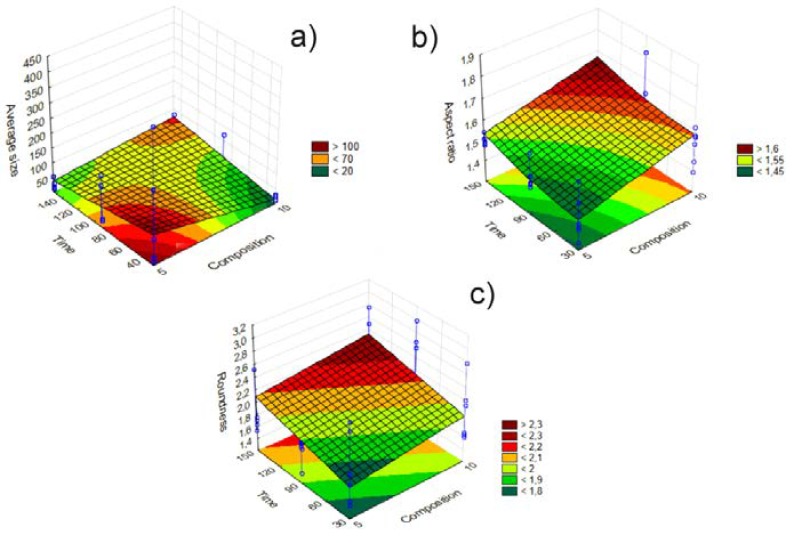
Surface plot diagrams of the SA products, investigating the effect of mixing time and solvent/antisolvent ratio on mean particle size, aspect ratio, and roundness; describing DS. Blue lines represent the standard deviation of the data. (**a**) represents the surface plot diagram of the mean particle size of the particles besides the modification of mixing time and solvent/antisolvent ratio (composition). (**b**) represents the surface plot diagram of the aspect ratio values besides the modification of mixing time and composition. (**c**) represents the surface plot diagram of roundness besides the modification of mixing time and solvent/antisolvent ratio (composition).

Surface plot diagrams were also taken (Figure 5) to obtain more information about the parameters that characterize the design space. Figure 5a shows the effects of changing the mixing time and the solvent/antisolvent ratio on mean particle size. Our DS is where lower mixing time values and composition ratios meet. This is true for Figure 5b, c, too. The application of lower mixing time and solvent/antisolvent values results in particles with lower aspect ratio and roundness values.

### 3.3. Polymorphism

**Figure 6:**
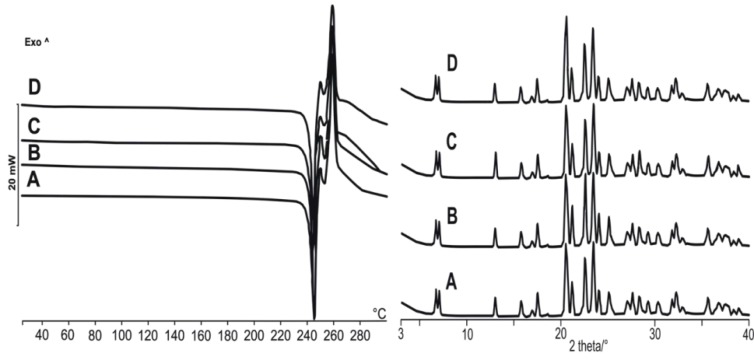
X-ray powder diffractometry (XRPD) and differential scanning calorimetry (DSC) results of the four products with proper mean particle size, aspect ratio, and roundness values.

After crystallization, it was necessary to verify that no polymorphic transitions had happened. For this reason, XRPD, DSC, and FTIR were used and it can be claimed that the crystalline material was of the same polymorphic form during the crystallization procedure (Figure 6). The thermal analysis shows that the melting points were the same between 245 and 246 °C and XRD patterns appeared at the same 2-theta values in each case.

**Figure 7:**
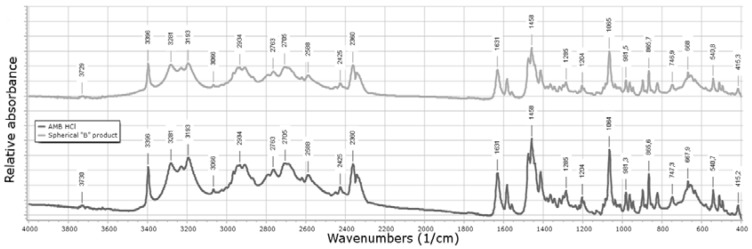
FTIR spectra of the original AMB HCl and the spherical agglomeration product (SAB).

FTIR spectra were also measured and those of the original material and our formulation were compared (Figure 7).

The FTIR of AMB HCl shows characteristic bands, for example, at 668 cm^−1^, 1631 cm^−1^, and 1285 cm^−1^ corresponding to the presence of functional groups such as aliphatic bromo compound, secondary amine, and secondary alcohol. All the other peaks appeared at the same wavenumber values, which mean that no undesirable reactions or polymorphic transitions happened during the crystallization. Although the sensitivity of the FTIR method is not as high as, for example, in the case of the GC method, it can be applied for the determination of the residual solvent content (RS) [47] and, based on our results, it can be claimed that the RS was under the detection limit. 

### 3.4. Light Microscopy Investigations

**Figure 8:**
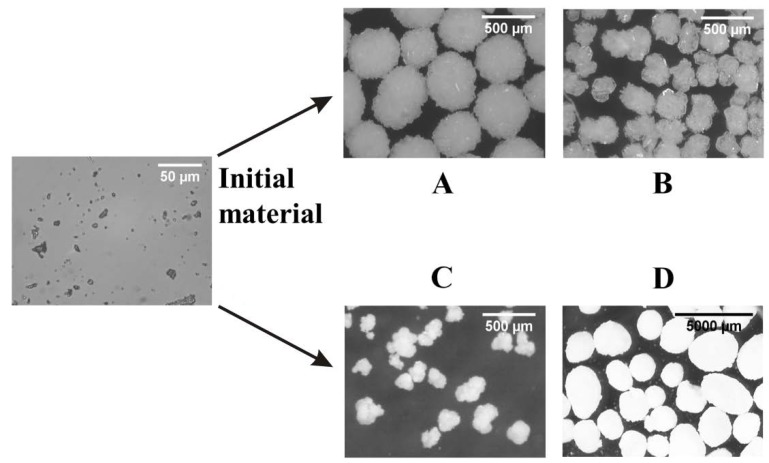
Light microscopy measurements of the initial material and the SA products produced with the application of different process parameters. (**A**) Light microscopic image of product SA (A); (**B)** light microscopic image of product SA (B); (**C**) light microscopic image of product SA (C); (**D**) light microscopic image of product SA (D). Process parameters are in Table 6.

During the statistical evaluation of the factorial design of the products, it became clear that four of them (A, B, C, D) had the correct mean particle size, aspect ratio, and roundness values compared to the target product. Mean particle size and roundness values were determined by light microscopy analysis. These values are shown in Table 6. Compared to the initial material, over an order of magnitude increase was reached and both aspect ratio and roundness values improved. Light microscopy images were taken in order to observe more closely the morphology of the particles. Exposures of the initial material and products A, B, C, and D are shown in Figure 8. 

These measurements confirmed that roundness values improved and the surface of the crystals became generally smoother (Figure 8A–D). Based on the light microscopy images, the SA method also enabled the production of uniform crystals.

### 3.5. Powder Rheology Tests

Product B was chosen for this experiment because it was produced in the highest amount (short mixing time, dT = 0 °C). Powder rheological properties were investigated and it was revealed that product A had far better flow properties; the Carr index and the Hausner ratio were also improved compared to the initial material (Table 7). This can ease the tableting process (for example, direct compression can be used instead of conventional tableting methods) and any other manipulations during the production (e.g., loading). Therefore, in the case of direct compression tablet making, the amount of the additives may be reduced. In Table 7, the improvements in the powder rheological properties are summarized.

### 3.6. Mechanical Strength Test

Mechanical strength tests were carried out in order to examine the mechanical stability of the crystalline material. The fracture curves of the products showed enough mechanical strength to maintain their spherical morphology with 2.53–3.13 N of fracture forces (Table 8), which is an especially high value regarding the mean particle size of the particles.

**Figure 9:**
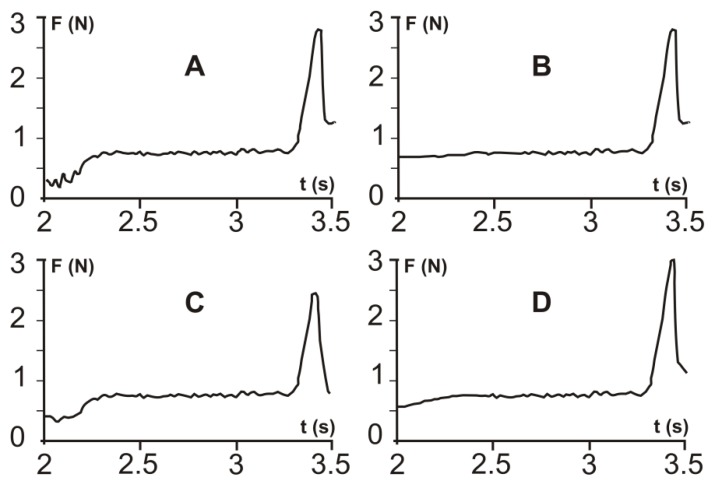
Representative fracture curves of the spherical agglomerates produced by the SA method.

The fracture curves of products A, B, C, and D are shown in Figure 9.

With a relatively long viscoelastic period, it can be claimed that the particles were resistant to slight mechanical impacts and only a larger force could break the particles. This is advantageous in the manufacture of a solid pharmaceutical formulation.

## 4. Discussion

The spherical agglomeration method was applied to the active agent AMB HCl in order to improve its powder rheological properties, which can ease the application of direct compression tablet making. The crystallization method was preceded by a risk assessment. The QTPPs, CQAs, CMAs, and CPPs were determined and the risk severity scores were evaluated by the Lean QbD program. According to the results, independent and dependent variables for a mixed 2–3 factorial design were determined and the factorial design was established. A total of 36 products were prepared and four of them (A, B, C, D) were marked as “good” for further investigations. During the product assay, it was first verified by X-ray powder diffraction (XRPD), thermal analysis (DSC), and infrared spectroscopy (FITR) that no polymorphic transitions had happened during the crystallization procedure. With light microscopy measurements, the mean particle size, roundness, and aspect ratio were examined and compared to the initial material. Since these parameters improved, it means that we had obtained smoother, larger, and more spherical crystals possessing improved powder rheological properties. Flow time, angle of repose, bulk density, and tapped density values were measured and the Carr index and the Hausner factor were calculated and compared to the initial material. A large improvement in these parameters was observed. In addition, the mean particle size became appropriate for direct compression. Larger particles have better flow properties due to their proportionally smaller surface area, which causes easier rolling on each other and decreased adhesion forces between the particles and tableting filling funnel. This is the topic of our next study. Mechanical strength characteristics were also investigated in order to predict whether the products would be suitable for pharmaceutical manipulations such as filling. It was revealed that the particles were stable and hard enough to keep their morphology constant during these procedures. In summarizing the main discoveries of the research, the spherical agglomeration crystallization process was applied successfully on the compound AMB HCl, resulting in spherical particles (140–450 µm) with smooth surfaces and improved powder rheological properties. The method was coupled with a risk assessment that was applied as part of the process development. It is a fairly individual and economical way for the production of AMB HCl crystals because it is based on the main impacts. The experiments were planned based on a factorial design and, therefore, less solid material and solvents were used compared to the original method, which starts with dozens of pre-experiments and applies an unpredictable method. Since the largest horizontal shakers on the market can only handle a maximum of 6 L of liquid, scale-up could be achieved but it is heavily dependent on the agitation type. It would be easier to implement with a propeller shaker but according to the current results, the morphology improved with the application of a horizontal shaker. The spherical crystallization could also be incorporated into the drug synthesis phase, which would significantly shorten the time necessary for manufacturing the pharmaceutical form because tablet making could be started from almost the same production line. Furthermore, by improving the morphology, the granulation process could be eliminated.

## Figures and Tables

**Table 1:** Comparison of the initial material and the target product.

	Initial Material	Target Product
**Mean particle size (number-based) (μm)**	13	80–1000
**Aspect ratio**	1.67	<1.5
**Roundness**	2.37	<1.5

**Table 2:** Quality target product profiles (QTPPs) of the spherical ambroxol hydrochloride (AMB HCl) produced by the spherical agglomeration (SA) method.

QTPP	Target	Justification
Direct compressible crystalline material	Direct compressible	Dosage form of this API is most commonly the tablet. Direct compression tablet making is a simple and fast method and is usable for active ingredients that are moisture sensitive [38].
Morphology	Larger, spherical particles	Larger, spherical particles possess better powder rheology parameters, which simplifies direct tablet making. Direct compression is an easy tableting method that avoids the long process of granulation [39].
Residual solvent content	The lowest possible, according to ICH guideline Q3C [40]	Residual solvent content is a critical parameter because of its toxic effect to the human body if its amount is higher than a certain described dose.
Habit	Spherical	Spherical crystals can easily roll on each other, which predestinate better flowing properties.
Powder rheology attributes	Good flowing properties	Better flowing properties simplify industrial operability of the powder [41].
Dosage form	Tablet	Tablet is the most common dosage form of this API [42,43].
Route of administration	GI tract	
Therapeutic effect	Expectoration improver	Ambroxol is a clinically proven systemically active mucolytic agent. When administered orally, the onset of action occurs after about 30 minutes [44].

**Table 3:** Critical quality attributes (CQAs) of the spherical crystal containing AMB HCl.

Quality Attributes	Target	Is it a CQA?	Justification
Physical attributes (color, odor, appearance)	White, odorless powder	No	Physical attributes are not directly related to patient safety; thus, it is not a critical attribute.
Particle size	Size range: 80–1000 µm	Yes	Particles of this size range are better applicable for direct compression in the case of a high-dose active agent since adherence and aggregation of the particles are uncharacteristic [45].
Particle size distribution	Narrow size distribution	Yes	Low span values indicate narrow particle size distribution, which makes the powder easy to process [45].
Roundness	≤1.50	Yes	This parameter is the measure of how closely the shape of an object approaches that of a mathematically perfect circle. Roundness applies in two dimensions, but it can refer to its three-dimensional analog, sphericity, as the image analyzer software works in 2D. In this way, spheres are treated as circles.
Aspect ratio	≤1.50	Yes	This parameter complements roundness very well. If these two are close to 1.00, it means that the investigated object is close to a sphere or circle.
Angle of repose	25–40° [46]	Yes	Angle of repose, flow time, Carr index, and Hausner factor values show the powder rheology attributes of the powder. Particles with “good” flow properties are suitable for direct tablet making.
Flow time	0–10 s [46]	Yes	
Carr index	1–25 [46]	Yes	
Hausner factor	1.00–1.34 [46]	Yes	

**Table 4:** The levels of the applied factors during the factorial design.

Factor	Levels			Number of Levels
	Low	Center	High	
Mixing type (qualitative)	horizontal shaker/marine propeller			2
Composition (solvent/antisolvent ratio)	1:5	-	1:10	2
dT (°C)	0	10	20	3
Mixing time (min)	30	90	150	3

**Table 5:** Parameters and effects causing significant changes in the dependent variables of the factorial design, applied for the SA method and the polynomial functions describing the conditions of significance.

Dependent Variable	Polynomial Function	R^2^	Adjusted R^2^
Mean particle size	y = 53.97 − 33.75x_1_ − 23.60x_2_ − 26.09x_3_ − 14.19x_4_ + 17.39x_4_^2^ + 26.83x_1_x_2_ + 30.32x_1_x_3_ + 17.35x_1_x_4_ − 15.20x_1_x_4_^2^ + 31.13x_2_x_3_ + 14.95x_2_x_4_ − 24.79x_2_x_4_^2^ − 23.68x_3_x_4_^2^	0.74	0.58
Aspect ratio	y = 1.508 − 0.032x_1_ + 0.032x_2_ + 0.013x_3_^2^ + 0.013x_4_^2^ − 0.021x_1_x_2_ − 0.040x_1_x_3_ − 0.016x_1_x_3_^2^ − 0.021x_2_x_4_ + 0.029x_1_x_4_^2^ + 0.021x_3_x_4_^2^ + 0.018x_3_^2^x_4_ + 0.022x_3_^2^x_4_^2^	0.61	0.41
Roundness	y = 1.9681 − 0.013x_1_ + 0.119x_2_ − 0.035x_3_ − 0.011x_4_^2^ − 0.228x_1_x_3_ + 0.095x_2_x_3_^2^	0.38	0.25
Yield	y = 38.94 − 0.19x_1_ + 1.46x_2_ + 9.25x_3_ + 0.12x_4_ + 5.66x_3_^2^ − 1.50x_4_^2^ − 3.90x_1_x_2_ − 10.47x_1_x_3_ − 3.33x_1_x_4_ − 3.56x_1_x_4_^2^ − 6.92x_2_x_3_	0.65	0.49
x_1_: Mixing type; x_2_: Composition; x_3_: Mixing time; x_4_: dT			

**Table 6:** Comparison of the initial material and the products made by the spherical agglomeration method (L = low-risk parameter; M = medium-risk parameter; H = high-risk parameter).

	Initial Material	A	B	C	D
**Mean particle size (µm)**	13.12	248.17	176.82	142.50	441.53
**Aspect ratio**	1.67	1.34	1.43	1.45	1.40
**Roundness**	2.37	1.41	1.37	1.88	1.49
**Yield (%)**	-	37.46	32.17	14.14	18.98
**Applied parameters**					
Mixing type	-	Horizontal shaker			
Composition	-	1:5			
Mixing time (min)	-	90 (M)	30 (L)	90 (M)	30 (L)
dT	-	0 (L)	0 (L)	10 (M)	10 (M)

**Table 7:** Improvements in the powder rheological properties comparing the initial material and the SA (B) product.

	Bulk Density (g/mL)	Tapped Density (g/mL)	Flow Time (s)	Angle of Repose (°)	Carr Index	Hausner Factor	Classification (Carr 1965)
Initial AMB HCl	0.54	0.81	unmeasurable	unmeasurable	32.67	1.49	Very poor
SA product (B)	0.43	0.53	13.6	31.3	18.8	1.23	Fair

**Table 8:** Mean values of the fracture forces of SA products based on 20 measurements.

Sample	Fracture Force (N)
A	2.95 ± 0.06
B	3.02 ± 0.04
C	2.53 ± 0.07
D	3.13 ± 0.04
